# Epidemic situation of the complex seasonality of imported influenza A and B virus transmission in Guangxi ports of China

**DOI:** 10.3906/sag-2008-63

**Published:** 2021-06-28

**Authors:** Xiangjuan LI, Zhongping LIANG, Jie GAN, Lingmin LU

**Affiliations:** 1 Guangxi International Travel Healthcare Center, Nanning Customs Port Clinic, Nanning, Guangxi China

**Keywords:** Epidemic, flu-imported situation, influenza, virus, travelers

## Abstract

**Background/aim:**

Analysis of the characteristics of influenza virus in imported cases in Guangxi province of China.

**Materials and methods:**

Throat swabs of imported cases with influenza-like symptoms were detected by real-time PCR from July 2016 to December 2019.

**Results:**

1292 laboratory detections of influenza were reported in 3974 influenza-like cases, of which 71.67% (926) were influenza A. The ratio of test positive was 32.82%. The proportion of detections of influenza B was 28.33% (366). A total of 70.51% of the cases mostly came from Vietnam (911). A total of 86.76% (1121) of the cases were imported from Dongxing Port, Nanning Airport, and Pingxiang Port. There was no statistical difference in all age groups. At the same time, 3 of the untyped A-type specimens were sequenced by next-generation sequencing. Among them, the sequences of 2 specimens from Vietnam had high homology with the influenza strain H3N2 in Hong Kong in 2017. The specimen sequence from Thailand is highly homologous to the influenza pandemic strain H1N1 in Brisbane, Australia in 2018.

**Conclusion:**

Imported influenza cases in Guangxi have occurred throughout the year, with higher numbers in winter and spring. The cases mostly came from Vietnam with influenza A. Relevant measures should be taken to control the further spread of the virus.

## 1. Introduction

Influenza viruses are associated with a substantial global burden of morbidity and mortality every year [1,2]. Influenza is a costly disease for pig producers and understanding its epidemiology is critical to control it [3]. Human influenza viruses can be divided into three types: A, B, and C according to the antigenicity of nuclear proteins [4]. The antigens of influenza A virus are prone to mutation, which often leads to influenza pandemics worldwide [5]. Influenza B virus has less antigenic variation with common sporadic epidemic, mainly causing small and medium epidemics in local areas [6]. Influenza C virus appears mainly in scattered form, affecting particularly infants and young children, and usually do not cause epidemics [7]. Influenza A and B viruses are the main pathogens causing human influenza [8,9]. In recent years, the influenza A epidemic has shown an increasing trend, and the local epidemic frequency caused by influenza A and B viruses has increased around the world [10,11].

In February 2017, Guangxi introduced the “Cross-Border Labor Cooperation Pilot Work Plan”, which fully leveraged the siphon effect of cheap labor in Vietnam’s border provinces and regions, met the growing border economic and trade needs, and further enhanced the competitiveness of Guangxi border areas. However, with the influx of Vietnamese labor force and the development of tourism, travel in Southeast Asian countries with strong exoticism has become a hot spot. Therefore, the transmission risk of imported sexually transmitted diseases in the border areas between China and Vietnam is thought to increase. Influenza A and B viruses are generally susceptible to the population, the number of high-risk groups is large, and there are many hidden infections carrying influenza viruses around the world. Thus, there is a large input and risk of epidemic.

The aim of the present study was to identify and compare the seasonality and epidemiological feature of seasonal influenza subtypes after the 2009A/H1N1 pandemic and to lay out a foundation for further investigation into the social and environmental factors affecting seasonal influenza transmission, providing guidance to develop a more specific strategy, and to strengthen the surveillance of infectious diseases at Guangxi ports.

## 2. Materials and methods

### 2.1. Subjects

Guangxi is located in the southwest of China. Guangxi is contiguous to Vietnam. The boundary line is about 637 km. It is the only province, which has 25 ports, including seaport, airport, and land port. From July 2016 to December 2019, from the immigration personnel at Guangxi Port, epidemiological investigations were conducted. Inclusion criteria were as follows: fever axillary temperature (37.5 °C), runny nose, stuffy nose, cough, headache, myalgia, fatigue, vomiting, and/or diarrhea. Exclusion criterion was self-declaration without cold symptoms or other discomfort symptoms. The pharyngeal swab samples were collected and saved at –40 °C to be tested.

### 2.2. Extraction and detection of nucleic acid 

The nucleic acid extraction adopts the automatic nucleic acid extraction instrument and matching reagents of American PerkinElmer Company (PerkinElmer, Inc., Waltham, MA USA), and the nucleic acid was extracted strictly in accordance with the instructions of the kit. Influenza virus detection kit (Guangzhou Daan Gene Biotechnology Co., Ltd.) was used to detect and type influenza virus by using the ABI7500 real-time fluorescent PCR instrument and real-time fluorescent RT-PCR method for port samples. The reagent instructions were strictly followed. It had no Ct value or Ct ≥ 38 and no obvious amplification curve, which was judged as negative. The value of Ct < 35 with obvious amplification curve was judged as positive. If Ct = 35–38, reexamination was performed once. If the result had obvious expansion curve, it was considered positive, otherwise it was considered negative.

**Sequence analysis**

At the same time, parts of the samples were sequenced with the Illumin II NextSeq 2000 sequencer, and the sequenced sequence was spliced, cleaned, and edited using cluster X software 3.0. MEGA 5 software was used to carry out the Neighbor-Joining tree, and the construction of the evolution tree was repeated 1000 times through the Interior Branch Test method to determine the reliability of its internal branches. 

## 3. Results

### 3.1. Laboratory testing

**Figure 1:**
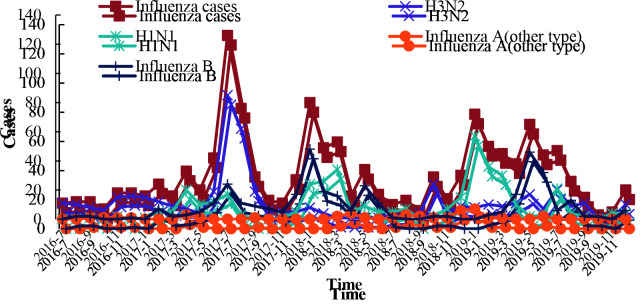
Time distribution of imported influenza positive cases at Guangxi port from 2016 to 2019.

A total of 3974 pharyngeal swab samples of influenza-like cases were collected, and the positive rate of influenza detection was 32.51% (1292 cases). Influenza A accounted for 71.67% (926 cases), and influenza B virus accounted for 28.33% (366 cases). Among them, H3N2 accounted for 38.00% (491 cases), H1N1 accounted for 30.50% (394 cases), and untyped A accounted for 3.17% (41 cases) (Table 1). A total of 4 imported influenza peaks occurred during the study period; the first influenza peak occurred in July 2017, mainly for H3N2; influenza peak entered the second peak period in January 2018, mainly for H1N1 and type B; influenza peak entered the third high peak period in 2019 January, mainly for H1N1, and influenza peak entered the fourth peak period, mainly for type B in May 2019 (Figure 1).

**Table 1:** Imported influenza cases at Guangxi ports from 2016 to 2019.

	Influenza-like cases	Influenza cases	Positive rate (%)	Proportion of influenza virus subtypes (cases) / influenza cases (%)			
				H1N1	H3N2	Unclassified influenza A	Influenza B
Total	3974	1292	32.51%	394/30.50%	491/38.00%	41/3.17%	366/28.33%

### 3.4. Age distribution

**Figure 2:**
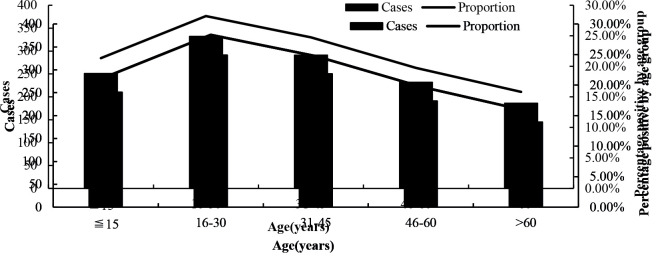
Number of cases and percentage of positive cases of imported influenza in various age groups at Guangxi port from July 2016 to December 2019.

Among the 1292 cases, there were 819 males and 473 females. Influenza cases were detected in five age groups, including <15, 16–30, 31–45, 46–60 and > 60 years old, indicating that different age have similar chance to have influenza (Figure 2). 

### 3.2. Origin of the case 

**Figure 3:**
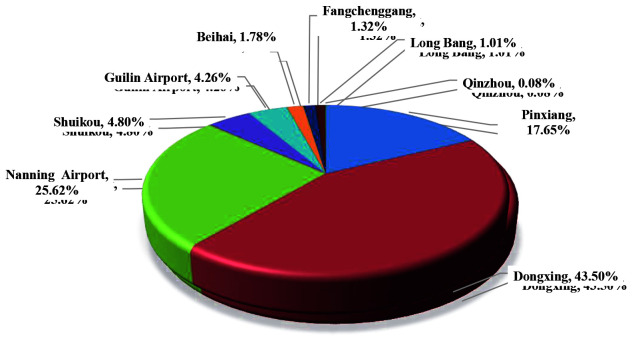
Sources of imported influenza cases at Guangxi ports.

Among the 1292 cases of influenza, in addition to the lack of information in 41 cases, the rest were mainly from 20 countries and regions including Association of Southeast Asian Nations (ASEAN) countries, Taiwan, Hong Kong, and South Korea. Among them, Vietnam had a maximum of 911 cases, accounting for 70.51% followed by Thailand 133 cases, accounting for 10.29%. ASEAN countries imported 1203 cases of influenza, accounting for 93.11%; nonASEAN countries and regions imported 61 cases of influenza, accounting for 4.72%. There were 33 cases mainly from in Taiwan, accounting for 2.55%. The principal influenza virus subtypes were H3N2 and H1N1 (Table 2 and Figure 3). 

**Table 2:** Sources of imported influenza cases at Guangxi ports from 2016 to 2019.

Sources	Vietnam	Thailand	Malaysia	Cambodia	Singapore	Indonesia	Myanmar	Laos	Philippines	Brunei	Taiwan	Hong Kong	South Korea	Other	Missing information
Influenza-like cases	3003	307	96	99	68	51	15	15	13	10	83	20	17	44	133
Influenza cases	911	133	50	34	29	24	9	5	3	5	33	6	5	17	28
H1N1	332	43	16	16	11	10	5	2	3	3	16	4	3	9	18
H3N2	268	46	23	11	12	6	1	2	0	1	12	2	2	3	5
Unclassified influenza A	29	5	0	3	1	1	0	0	0	0	0	0	0	2	0
Influenza B	282	39	11	4	5	7	3	1	0	1	5	0	0	3	5

### 3.3. Case entry port

**Figure 4:**
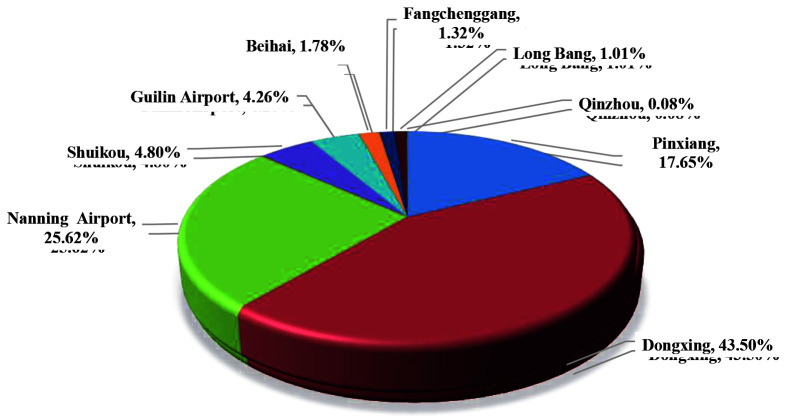
Distribution of imported influenza cases at Guangxi ports.

Influenza cases were mainly imported from Dongxing Port, which consist of a total of 562 cases, accounting for 43.50%; it was followed by Nanning Airport Port with 331 cases, accounting for 25.62%. Influenza cases in Pingxiang Port were 228, accounting for 17.65%. The influenza cases detected at these three ports accounted for 86.76% of the total imported influenza cases at Guangxi Port (Table 3 and Figure 4). 

**Table 3:** Distribution of imported influenza cases at Guangxi ports from 2016 to 2019.

Input port	Pingxiang	Dongxing	Nanning Airport	Shuikou	Guilin Airport	Beihai	Fangchenggang	Long Bang	Qinzhou
Influenza-like cases	1479	1113	898	216	104	81	48	15	20
Influenza cases	228	562	331	62	55	23	17	13	1

### 3.5. Influenza virus gene sequence analysis and homology comparison 

**Figure 5:**
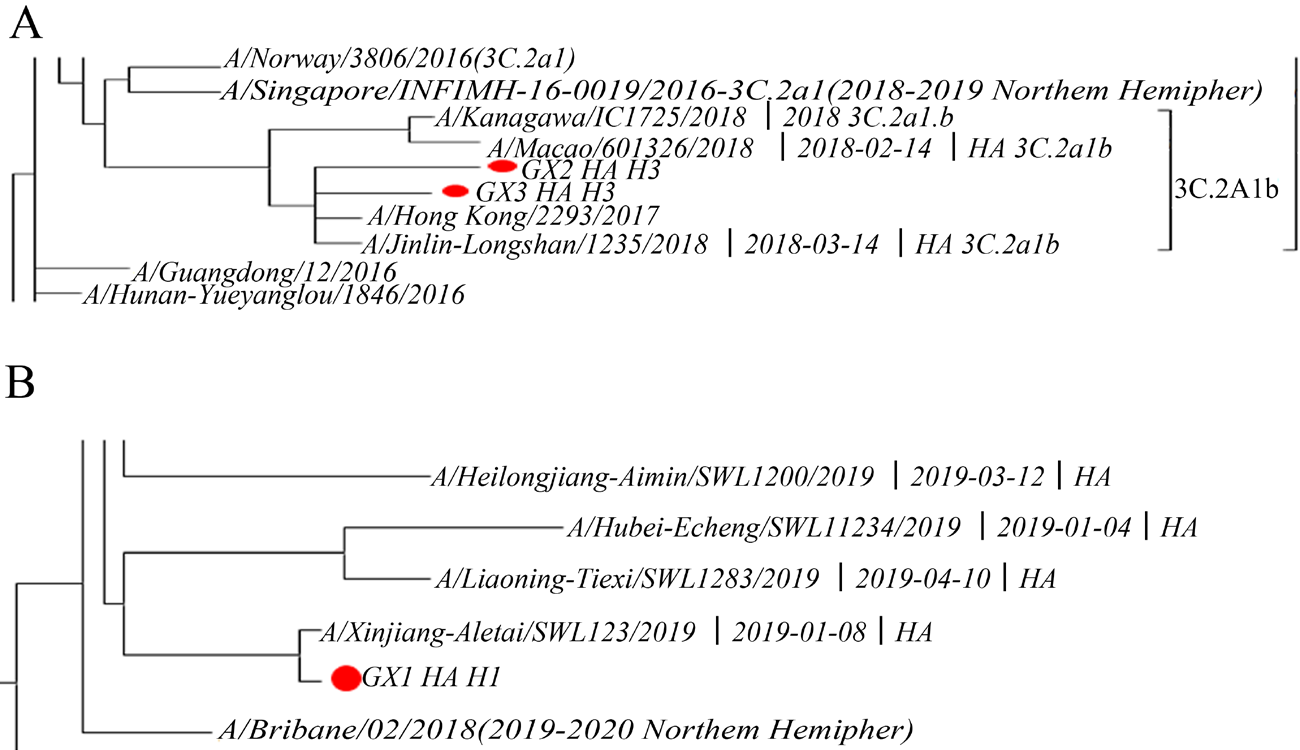
Phylogenetic tree analysis of influenza virus sequences from Vietnamese patient specimens (2 copies) (A). Phylogenetic tree analysis of influenza virus sequence of patients entering Thailand (B).

In this study, three of the untyped influenza A virus specimens were sequenced by whole genome. Among them, the sequences of two specimens from Vietnam had high homology with the 2017 H3N2 pandemic strain emerged in Hong Kong (Figure 5A). The sequence of a specimen from Thailand was highly homologous to the H1N1 pandemic strain emerged in Brisbane, Australia in 2018 (Figure 5B).

## 4. Discussion

The zoonotic viruses can emerge unexpectedly in nature resulting in the establishment of viral infections with important economic and health burden [12]. Influenza viruses infect many vertebrates, as influenza A, B, and C viruses infecting humans, and their high mutation rates prevent immunity. [13]. While the early start and higher intensity of the 2012/13 influenza A virus epidemic was not unprecedented, it was the first influenza A virus epidemic season since the 2009 H1N1 influenza pandemic where the H3N2 subtype predominated [14]. Influenza A and B fluctuated simultaneously or alternately. This was consistent with the global influenza trend in recent years and the local influenza situation [15–17]. Influenza A and B viruses can infect people of different ages, and children and adolescents were at high-risk for influenza A or B virus infection [18]. In this study, 70.51% (911) of the confirmed cases of influenza were mainly from Vietnam, so Vietnamese entry personnel were the key surveillance objects of influenza. The imported influenza cases were mainly influenza A virus infection in winter and spring, and influenza B virus was distributed. The influenza cases were detected in all age groups, which was consistent with previous studies.

A universal influenza vaccine could considerably alleviate the public health burden of both seasonal and pandemic influenza [19]. The effect of influenza vaccine was closely related to the degree of matching of vaccine strains and epidemic strains [20, 21]. Whole-genome sequencing could provide a faster and more reliable method for outbreak monitoring and supplement routine infection prevention and control team work to allow the prevention of transmission [22]. Therefore, strengthening the clinical and epidemiological monitoring of influenza virus infection at ports was conducive to the early detection of mutant strains, scientific assessment of their occurrence risks, epidemic trends, and potential risk areas to better prevent and control the international spread of influenza, the prevention, and control of influenza in Guangxi ports, which provided a theoretical basis for molecular epidemiology. At the same time, we should strengthen the port health and quarantine line of defense to give full play to the frontier health and quarantine responsibilities of preventing the infectious diseases from entering and leaving. 

## 5. Conclusion

This study highlights the complexity of imported influenza virus activity in a subtropical region of southwest China and suggests that the epidemic features of influenza A and B vary by subtype and over time. The current one-season vaccination in Guangxi, China should be carefully reconsidered.
